# High lethality and minimal variation after acute self-poisoning with carbamate insecticides in Sri Lanka – implications for global suicide prevention

**DOI:** 10.1080/15563650.2016.1187735

**Published:** 2016-06-02

**Authors:** Thomas Lamb, Liza R. Selvarajah, Fahim Mohamed, Shaluka Jayamanne, Indika Gawarammana, Ahmed Mostafa, Nicholas A. Buckley, Michael S. Roberts, Michael Eddleston

**Affiliations:** ^a^ Department of Pharmacology, Toxicology, and Therapeutics, University/BHF Centre for Cardiovascular Science, University of Edinburgh, Edinburgh, UK; ^b^ South Asian Clinical Toxicology Research Collaboration, Department of Clinical Medicine, University of Peradeniya, Peradeniya, Sri Lanka; ^c^ Department of Pharmacology, School of Medical Sciences, University of Sydney, Sydney, Australia; ^d^ Department of Medicine, University of Kelaniya, Kelaniya, Sri Lanka; ^e^ Therapeutics Research Centre, School of Medicine, University of Queensland, Brisbane, Australia; ^f^ Department of Pharmaceutical Chemistry, Faculty of Pharmacy, Helwan University, Helwan, Egypt

**Keywords:** Lung, pesticide poisoning, suicide

## Abstract

**Background:** Highly hazardous organophosphorus (OP) insecticides are responsible for most pesticide poisoning deaths. As they are removed from agricultural practice, they are often replaced by carbamate insecticides of perceived lower toxicity. However, relatively little is known about poisoning with these insecticides.

**Methods:** We prospectively studied 1288 patients self-poisoned with carbamate insecticides admitted to six Sri Lankan hospitals. Clinical outcomes were recorded for each patient and plasma carbamate concentration measured in a sample to confirm the carbamate ingested.

**Findings:** Patients had ingested 3% carbofuran powder (719), carbosulfan EC25 liquid (25% w/v, 389), or fenobucarb EC50 liquid (50% w/v, 127) formulations, carbamate insecticides of WHO Toxicity Classes Ib, II, and II, respectively. Intubation and ventilation was required for 183 (14.2%) patients while 71 (5.5%) died. Compared with carbofuran, poisoning with carbosulfan or fenobucarb was associated with significantly higher risk of death [carbofuran 2.2%; carbosulfan 11.1%, OR 5.5 (95% CI 3.0–9.8); fenobucarb 6.3%, OR 3.0 (1.2–7.1)] and intubation [carbofuran 6.1%; carbosulfan 27.0%, OR 5.7 (3.9–8.3); fenobucarb 18.9%, OR 3.6 (2.1–6.1)]. The clinical presentation and cause of death did not differ markedly between carbamates. Median time to death was similar: carbofuran 42.3 h (IQR 5.5–67.3), carbosulfan 21.3 h (11.5–71.3), and fenobucarb 25.3 h (17.3–72.1) (*p* = 0.99); no patients showed delayed onset of toxicity akin to the intermediate syndrome seen after OP insecticide poisoning. For survivors, median duration of intubation was 67.8 h (IQR 27.5–118.8) with no difference in duration between carbamates. Reduced GCS at presentation was associated with worse outcome although some patients with carbosulfan died after presentation with normal GCS.

**Conclusions:** We did not find carbamate insecticide self-poisoning to vary markedly according to the carbamate ingested although the case fatality varied according to the concentration and formulation of the insecticide. Carbamate poisoning did not appear to be much less toxic than poisoning with some liquid OP insecticide formulations, e.g., chlorpyrifos EC40, that we have previously noted in these same hospitals (Lancet 2005, 366:1452–1459; QJM 2006, 99:513–522). Replacement of WHO Class II Toxicity OP insecticides in agriculture with high-strength liquid carbamate formulations may not substantially reduce case fatality after pesticide poisoning and, therefore, global suicide rates.

## Introduction

Pesticide self-poisoning is a major global health problem, with hundreds of thousands of deaths each year.[1–4] The majority of deaths over the last 30 years have been due to organophosphorus (OP) insecticide poisoning. However, recent pesticide regulations in for example Sri Lanka [5,6] and China [7,8] has resulted in reduced agricultural use of highly hazardous OP insecticides and a shift towards the use of other insecticides, including carbamates.

Like OP insecticides, carbamates inhibit the acetylcholinesterase (AChE) enzyme resulting in overstimulation of cholinergic synapses and death from acute respiratory failure.[9,10] However, unlike many OP insecticides, carbamates do not require activation after absorption and onset of clinical features can be rapid.[11,12] Management involves resuscitation, ventilatory support, and administration of atropine;[13,14] oximes are usually not recommended in carbamate poisoning due to rapid spontaneous reactivation of AChE.[10]

**Figure 1.**
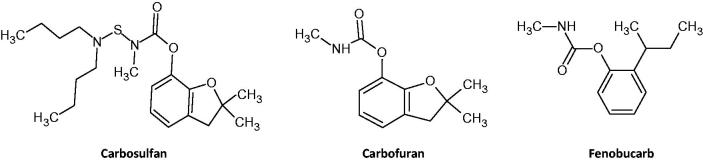
Structures of the three carbamate insecticides. Carbosulfan is metabolised to carbofuran.

The three carbamate insecticides used locally in Sri Lankan agriculture are carbofuran, carbosulfan, and fenobucarb (BPMC) (Table 1 and Figure 1). Their formulation varies, secondary to their rat oral toxicity and Sri Lankan pesticide legislation. Carbofuran is a WHO Class Ib toxicity (‘highly hazardous’) insecticide that is only permitted in Sri Lanka as a 3% wettable powder. In contrast, both carbosulfan (a pro-poison of carbofuran) and fenobucarb are WHO Class II toxicity (‘moderately hazardous’) insecticides that are available in liquid formulations as 25% and 50% emulsifiable concentrates (EC), respectively (again reflective of their relative rat oral toxicity – Table 1).

**Table 1.** Characteristics of the three carbamate insecticides.

Carbamate insecticide	Carbofuran	Carbosulfan	Fenobucarb
CAS number	1563-66-2	55285-14-8	3766-81-2
WHO toxicity class	Ib Highly hazardous	II Moderately hazardous	II Moderately hazardous
Rat oral LD_50_^a^			
– WHO [30]	8	250	620
– CPH [31]	8	209	640
Fat solubility (log *p*)^b^	2.32	5.57	2.78
Preparation	Solid	Liquid	Liquid
Formulation			
– g/kg or g/L	30	250	500
– Size/volume	0.5–1.0 kg	100–200 mL	100–200 mL

^a^ Two sources of rat oral LD_50_ values (mg/kg) are given here. Abbreviations: CPH: Crop Protection Handbook; WHO: World Health Organization.

^b^ Log *P* (or log *K*_ow_), the logarithm of the partition coefficient between n-octanol and water, correlates with fat solubility. The values given here are from TOXNET: ChemIDplus, US National Library of Medicine. A value of <1.0 indicates a hydrophilic compound; a value >4.0 indicates a highly fat soluble compound.

Many texts consider carbamate poisoning to be similar to, but less severe than, OP insecticide poisoning and prolonged ventilation rarely necessary.[9,10,12,14] However, there is relatively little high-quality published clinical data on carbamate poisoning with no large prospective case series of multiple compounds. We do not know whether there is marked variation in lethality and clinical syndrome between different carbamate insecticides, as occurs importantly with OP insecticides.[15,16]

To answer this question, we established a prospective cohort of Sri Lankan patients acutely self-poisoned with carbamates and compared the results with a case series of patients with insecticide poisoning treated at the same time in the same hospitals.[16–18]

## Methods

### Patients

Patients were seen on admission to six Sri Lankan hospitals as part of an ongoing cohort study of acute self-poisoning that started in March 2002 in Anuradhapura, June 2002 in Polonnaruwa, November 2002 in Kurunegala, and September 2006 in Chilaw, Galle, and Peradeniya. The last patient was recruited to this specific cohort in October 2010. The great majority of patients were exposed due to self-harm, rather than unintentional or occupational exposure. In a series of 1100 pesticide poisoning patients seen in these hospitals during 2002–2003, 93.8% had poisoned themselves.[19]

Patients were included in this study if they had a history of carbamate ingestion as indicated by the patient or relatives, the transferring doctor, or the pesticide bottle. Patients who ingested more than one carbamate or other poison (except for alcohol) were excluded from the study. Patients who were reported to have ingested a carbamate but for which the specific compound was unknown were grouped as ‘unknown carbamate’.

Patients remained under the care of the consultant physicians. Management protocols were agreed between the ward doctors and the study team. Decisions about intubation and transfer of patients to intensive care were made by the medical team independently of study doctors. All decisions were made on the basis of the patient’s clinical condition and available resources, not on the particular carbamate ingested.

Atropine was administered following a standard protocol.[13,16] Once resuscitated, until 16 October 2004, patients or their relatives were approached concerning recruitment to a RCT of activated charcoal that was nested into the cohort;[20] informed signed consent was obtained from patients or relatives.

All patients were seen regularly by full time study doctors at least every 3 h and more according to clinical need. Significant events (intubation, seizures, and death) were recorded at the time of the event. Patients were also seen on a study ward round twice each day and their condition over the previous 12 h recorded.

Patients were first managed on the medical ward. Seriously ill patients, as judged by the ward’s medical staff, were transferred to the intensive care unit (ICU) as beds became available. Each hospital had 2–8 ICU beds for medical patients; there was always difficulty in obtaining a bed. Criteria for intubation were tidal volume less than 180 ml/breath using Wright’s respirometer, respiratory rate <10 breaths/min, or failure of a Guedel airway to preserve airway function. Arterial blood gases were not available to guide therapy. Hypotensive patients, not responding to atropine and fluid resuscitation, were treated with dopamine and dobutamine as necessary by infusion pump. Norepinephrine and epinephrine infusions were not used; bolus epinephrine was administered for cardiac arrests. Ethics approval was obtained from Oxfordshire Clinical Research Ethics Committee and Faculty of Medicine Ethics Committee, Colombo.

### Toxicological analysis

Blood samples were taken from a nested sample of patients recruited to the RCT until December 2003 and used to test the accuracy of the history of carbamate ingestion. Admission plasma samples (taken a median time of 4–5 h post-ingestion for all three carbamates) were assayed in Brisbane for carbamate concentration in 198 patients (96 carbofuran, 75 carbosulfan, and 27 fenobucarb) by liquid chromatography/tandem mass spectrometry, as published.[21] The limits of detection were 10 ng/mL for carbosulfan and fenobucarb and 20 ng/mL for carbofuran. Neither butyrylcholinesterase nor acetylcholinesterase were measured in these samples.

### Statistics

Primary data analysis was performed on Graphpad Prism 5 (Graphpad Inc., San Diego, CA). Demographic factors and clinical characteristics were summarised using counts for categorical variables and the median (interquartile range [IQR]) for non-normally distributed continuous variables. Case fatality and need for intubation in the carbosulfan and fenobucarb groups were calculated and compared with carbofuran by calculating odds ratios and 95% CI.

## Results

Between March 2002 and October 2010, 1288 patients with acute self-poisoning were identified as ingesting a carbamate insecticide. Of these, 719 (55.8%) had ingested carbofuran, 389 (30.2%) carbosulfan, 127 (9.9%) fenobucarb, and 53 (4.1%) an unspecified carbamate. Demographic data for the three carbamate patient groups were similar at baseline (Table 2).

**Table 2.** Demographic and admission characteristics following carbamate self-poisoning.

Carbamate insecticide	Carbofuran (*n* = 719)	Carbosulfan (*n* = 389)	Fenobucarb (*n* = 127)
*Demographics*			
Male [*n* (%)]	428 (59.5)	274 (70.4)	82 (64.6)
Age [years, median (interquartile range [IQR])]	31 (21–42)	28 (20–38)	27 (20–37)
Time to presentation [h; median, IQR]^a^	5 (3–8)	4 (3–7)	4 (2–6)
Activated charcoal treatment			
No charcoal (%)	42.2	46.0	42.7
Single dose (%)	46.1	43.4	40.3
Multiple dose (%)	11.7	8.6	8.9
*Admission characteristics*			
Glasgow Coma Score (GCS) [median (IQR)]	15 (15–15)	15 (11–15)	15 (13–15)
Carbamate plasma concentration [mcg/L; median, (IQR)]^b^	53.9 [0–272]	18.3 [0–44.5]	1160 [133–47,500]

^a^ The time of ingestion was known for 702, 375, and 124 patients, respectively.

^b^ The carbamate concentration was measured in 96 carbofuran patients, 75 carbosulfan patients, and 27 fenobucarb patients.

### Plasma pesticide concentrations

We measured the concentration of the carbamate active ingredients (both parent and metabolite in the case of carbosulfan poisoning) in a nested sample of patients to estimate the accuracy of the history of ingestion and compare plasma concentrations. Carbamates were identified in plasma in 167/198 (84.3%) cases (carbofuran 76/96 [79.2%], carbosulfan 65/75 [86.7%], and fenobucarb 26/27 [96.3%]).

The differences in the formulation (and, therefore, the ease of ingestion) and rapid metabolism of carbosulfan to carbofuran was notable in the median plasma carbamate concentrations of patients poisoned by the three pesticides taken a median of 4–5 h post-ingestion (Table 2). Carbofuran, as a metabolite formed by CYP3A4 [22] from the relatively high concentration carbosulfan EC25 formulation, was present at higher concentrations in plasma of patients with carbosulfan poisoning (median 285 [IQR 0–1404] mcg/L) than after carbofuran poisoning (53.9 [IQR 0–272] mcg/L, *p* < 0.019) despite similar median times to presentation and blood sampling.

### Clinical variation

Of the 1288 patients, 183 (14.2%) patients required intubation and ventilation and 71 (5.5%) died. The differences in the formulation, amount ingested, and plasma concentration, were reflected in the variation of case fatality and need for ventilation among the patient groups (Table 3).

**Table 3.** Outcomes following admission to hospital with carbamate insecticide self-poisoning.

Carbamate insecticide	Carbofuran (*n* = 719)	Carbosulfan (*n* = 389)	Fenobucarb (*n* = 127)
*Outcomes*			
Number of deaths (*n*)	16	43	8
Case fatality ratio [% (95% CI)]	2.2 (1.3–3.6)	11.1 (8.1–14.6)	6.3 (2.8–12.0)
Time to death (h; median, IQR)	42.3 (5.5–67.3)	21.3 (11.5–71.3)	25.3 (17.3–72.1)
Number requiring intubation (*n*)	44	105	24
Proportion [% (95% CI)]	6.1 (4.5–8.1)	27.0 (22.6–31.7)	18.9 (12.5–26.8)
Time to intubation (h; median, IQR)	3.0 (1.5–5.9)	3.4 (1.8–10.5)	3.8 (2.0–6.5)
Duration of intubation (survivors) (h; median, IQR)	54.3 (12.3–90.0)	70.8 (36.3–132.9)	79.5 (34.3–158.0)
Number with seizures (*n*)	4	8	1
Proportion [% (95% CI)]	0.6 (0.2–1.4)	2.1 (0.9–4.0)	0.8 (0.0–4.3)

Carbosulfan and fenobucarb poisoning was more severe than carbofuran poisoning: the case fatality for carbofuran was 2.2%, carbosulfan 11.1% (odds ratio [OR] 5.5 [3.0–9.8], *p* < 0.0001 versus carbofuran), and fenobucarb 6.3% (OR 3.0 [1.2–7.1] *p* < 0.018 versus carbofuran, Table 3). Of the patients ingesting an unknown carbamate, 7.5% died.

**Figure 2.**
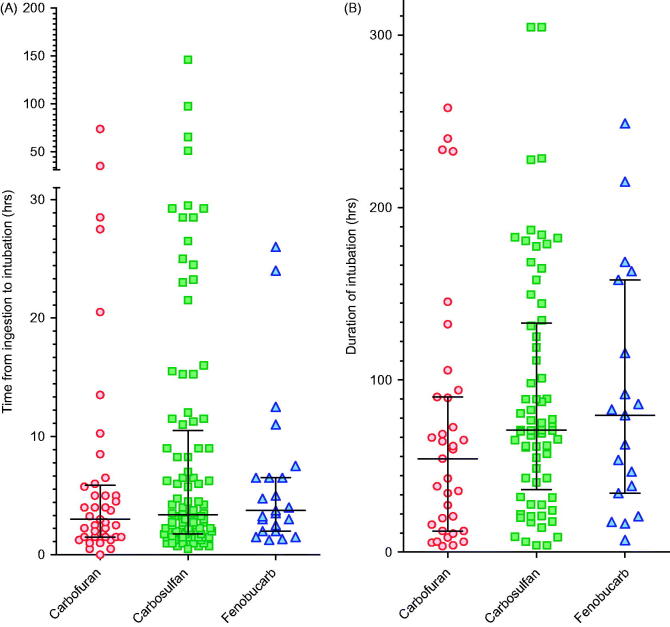
Time to (A) first intubation and (B) for survivors, duration of intubation, according to carbamate ingested. There was little difference in time to intubation and duration of intubation between pesticides. The majority of patients were intubated between 0.5 and 5 h post-ingestion; nearly all were intubated within 48 h. Patients intubated after 48 h were typically intubated for worsening pneumonia and not for peripheral neuromuscular failure as noted after OP insecticide poisoning. Bars show the median (IQR) time.

The need for intubation and ventilation was more common with carbosulfan and fenobucarb (Table 3), the proportion of patients requiring intubation with carbofuran being 6.1%, carbosulfan 27.0% (OR 5.7 [3.9–8.3] *p* < 0.0001 versus carbofuran), and fenobucarb 18.9% (OR 3.6 [2.1–6.1] *p* < 0.0001 versus carbofuran, Table 3). Of patients ingesting an unknown carbamate, 18.9% required intubation. The time to intubation post-exposure did not differ significantly between carbamates (Table 3 and Figure 2(A)).

**Figure 3.**
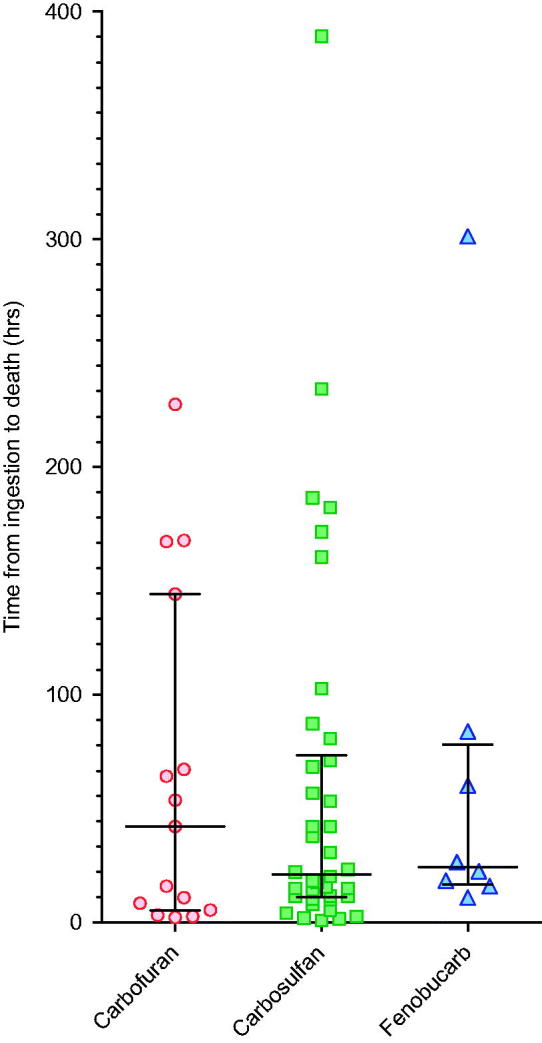
Time between ingestion and death for patients poisoned by the three carbamates. Paired times of ingestion and death were available for 15/16 carbofuran, 38/43 carbosulfan, and 8/8 fenobucarb fatal cases. Bars show the median (IQR) time.

Unlike OP insecticides,[16] the pattern of toxicity and death (as distinct from severity) did not differ markedly between the individual carbamates. None of the carbamates typically produced a delayed onset of toxicity as is common after poisoning with the fat soluble OP, fenthion, despite the octanol–water partition coefficient (log *K*_ow_, a marker of water solubility) of carbosulfan being high (Table 1). Of the patients who died for whom the time of ingestion was known (*n* = 65), most deaths (41/65, 63.1%) occurred within the first 48 h after ingestion (Figure 3). A few deaths from carbofuran (4/15, 26.7%), carbosulfan (6/38, 15.8%), or fenobucarb (1/8, 12.5%) occurred late, after 5 d, due to complications of long-term ventilation or the respiratory or neurological complications of events that occurred before admission. The median time to death for patients poisoned by the three pesticides did not differ between carbamate insecticides (Table 3). Overt seizures were rare for all three carbamates, occurring in just 14/1288 cases (1.1%, Table 3).

### Duration of ventilation

Of the intubated patients (183) for whom the times of ingestion and of intubation were known (156, 85.2%), the majority (125/156; 80.1%) were intubated within 12 h of ingestion (Figure 2(A)) because of cholinergic features and respiratory failure. Only 31 (19.9%) were intubated after 12 h.

Of these 156 intubated patients, 38 (24.4%) died before hospital discharge. Six (15.8%) died within 8 h of ingestion from cardiovascular instability – each patient was severely ill on admission, was never stabilised, and did not regain consciousness before death. Sixteen (42.1%) died 12–48 h after ingestion; all but one remained unconscious until death. Sixteen (42.1%) died later during their inpatient stay, from 2 to 9 d post-intubation, clinically due not to acute toxicity but to late complications of poisoning (pneumonia, pre-hospital aspiration, and/or ventilation) (post-mortem data were not available).

For survivors, the median duration of intubation was 67.8 (IQR 27.5–118.8) h. There was no significant difference in duration among carbamates: carbofuran 54.3 (12.3–90.0) h, carbosulfan 70.8 (36.3–132.9) h, and fenobucarb 79.5 (34.3–158.0) h (Figure 2(B)).

After OP insecticide poisoning, we found that patients who were intubated late, more than 24 h after ingestion, required longer periods of intubation and ventilation (early 33 versus 219 h, *p* < 0.001).[17] To determine whether such an effect was seen with carbamate poisoning, we compared the duration of intubation in patients intubated for the first time before and after 24 h.

**Figure 4.**
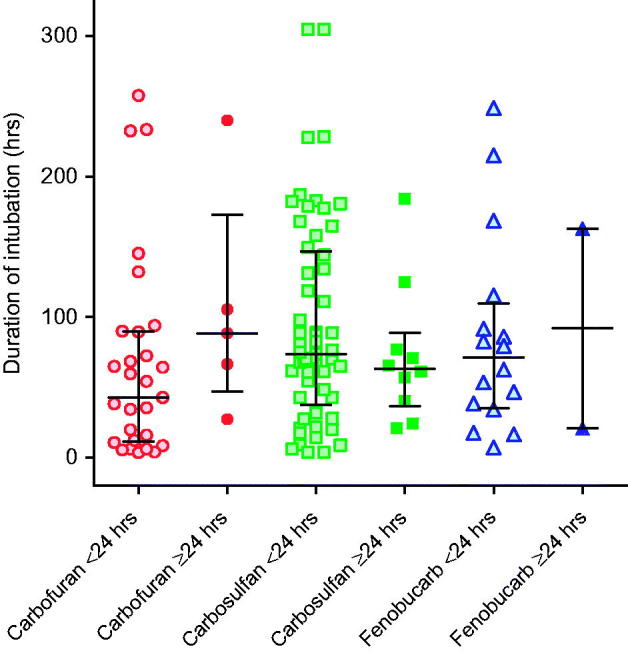
Comparison of time to first intubation with need for ventilation. Patients intubated within 24 h of admission did not show a shorter duration of intubation compared to patients intubated after 24 h. Bars show the median (IQR) time.

We found no such effect in carbamate-poisoned patients (Figure 4), suggesting that sustained neuromuscular junction (NMJ) dysfunction is not a common feature of carbamate poisoning. Of the 183 intubated patients, 138 (75.4%) survived to discharge; 119 (86.2%) had ingested one of carbofuran, carbosulfan, or fenobucarb. The median duration of intubation according to time of first intubation (early <24 h versus late ≥24 h) did not differ for any of the three insecticides: carbofuran early 42.8 h (IQR 11.5–89.8) versus late 88.5 h (IQR 46.9–173, *p* = 0.145); carbosulfan early 73.8 h (IQR 37.4–147) versus late 63.4 h (IQR 36.4–89.0, *p* = 0.471); fenobucarb early 71.0 h (IQR 35.3–110) versus late 91.9 h (IQR 20.8–163, *p* = 0.943). Only 23/138 patients (16.7%) were intubated for longer than 1 week.

### GCS on admission and prognosis

A reduced Glasgow coma score (GCS) was a predictor of poor prognosis. Compared with patients presenting with a GCS of 15/15 who had a case fatality of 16/942 (1.7%), all patients with reduced consciousness GCS ≤14/15 or with profound coma (GCS 3/15) had case fatalities of 54/332 (16.3%, OR 11.3 [6.3–20.0], *p*= <0.001) or 32/69 (46.4%, OR 50.1 [25.3–99.3], *p*= <0.001), respectively.

**Figure 5.**
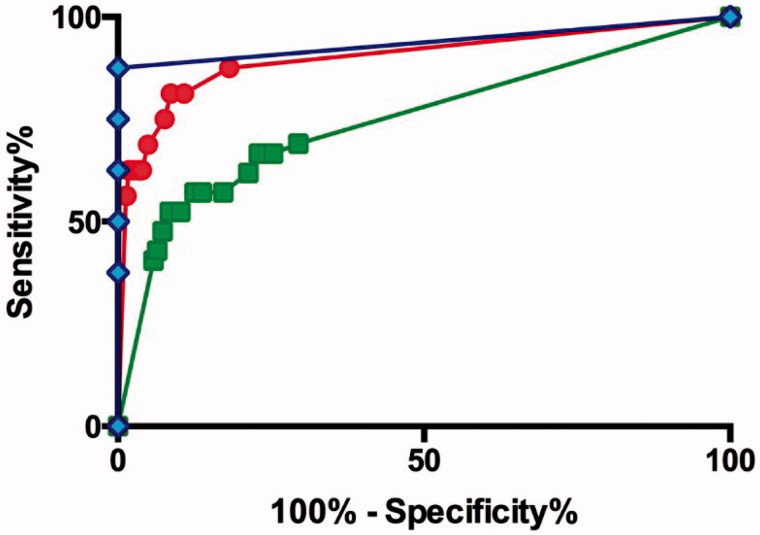
Receiver operated curve analysis for the carbamates. Blue diamonds: fenobucarb; red circles: carbofuran; green squares: carbosulfan.

Fatal cases with carbofuran, carbosulfan, and fenobucarb poisoning presented with median (IQR) GCS scores of 3 (3–11), 6 (3–15), and 8 (3–12), respectively. Although a GCS of 15 was overall a good prognostic indicator, it varied between carbamate. For patients who had ingested carbofuran with a GCS of 15 at presentation, the case fatality was 2/570 (0.4%), compared with that of carbosulfan 13/256 (5.1%, OR 15.2 [3.4–67.9] *p*= <0.001) and fenobucarb 1/85 (1.2%, OR 3.3 [0.30–37.3] *p* = 0.344). Receiver operated curve (ROC) analysis for the three carbamates showed a better sensitivity and specificity for fenobucarb (AUC 0.94 [CI 0.80–1.07]) compared with the other carbamates and all carbamates together (AUCs: carbofuran 0.90 [CI 0.80–1.00], carbosulfan 0.75 [CI 0.66–0.84], all carbamates 0.82 [CI 0.76–0.88]) (Figure 5).

## Discussion

Pesticide self-poisoning causes hundreds of thousands of deaths each year.[3] OP insecticides have long been responsible for the majority of these deaths but the most toxic OP compounds are now slowly being removed from agricultural practice. In the developing world, they are being replaced by carbamates and neonicotinoids together with other more modern insecticides. Neonicotinoids, such as imidacloprid, and other modern insecticides are less toxic [18]; however, the effect of shifting to carbamates on deaths from pesticide self-poisoning is unclear.

Although carbamates are generally considered to be less toxic than OP insecticides, we show here that poisoning with liquid EC formulations of WHO Class II carbamates results in case fatalities similar to many WHO Class II OP insecticides. A similar conclusion was drawn for Class I OP and carbamate insecticides from a small retrospective South Korean study comparing poisoning by the carbamate methomyl (17 cases) with poisoning by the OP insecticides methidathion, ethyl 4-nitrophenyl phenylphosphonothioate (EPN), dichlorvos, phosphamidon, and parathion (42 cases).[23] Switching from OP liquid formulations to high-concentration carbamate liquid formulations will likely not have a major effect on global suicide rates.

We found marked differences in plasma concentration of the different carbamates, with fenobucarb having significantly higher blood concentrations, due to the higher concentration liquid formulation. This high concentration was not associated with a high fatality due to the inherently lower toxicity of this carbamate compared with carbofuran.

The plasma carbofuran concentration was higher in patients ingesting carbosulfan than those ingesting carbofuran, due to the more concentrated liquid formulation of carbosulfan and the conversion of carbosulfan to carbofuran by CYP3A4 metabolism. *In vitro* studies indicate that polymorphisms in this CYP enzyme [24] may result in varied production of the toxic metabolite.[22] However, we were not able to measure CYP3A4 activity in these patients to assess the importance of polymorphisms in toxicity.

**Figure 6.**
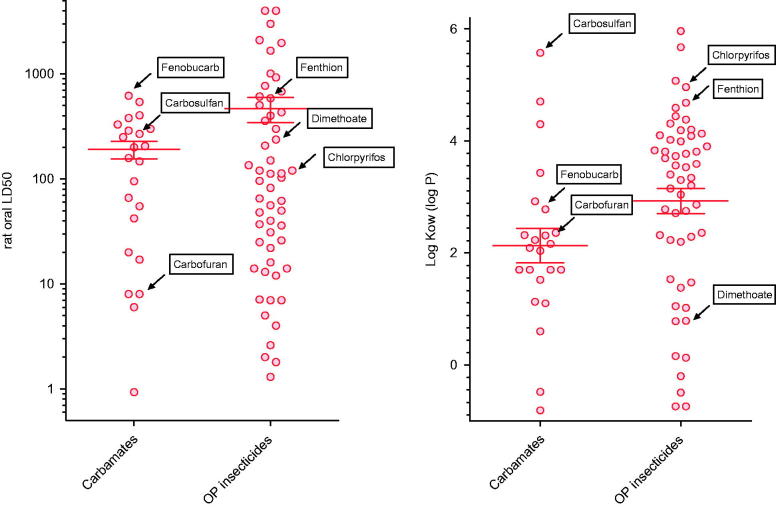
Rat oral toxicity (LD50) and octanol/water solubility (log *K*_ow_) for the three carbamates, compared to all carbamates and all OP insecticides. Data on rat oral toxicity were taken from the WHO Classification of Pesticides; data on log *K*_ow_ from NLM TOXNET. The three WHO Class II OP insecticides most closely studied in these same hospitals [16] are also indicated.

Although the three most commonly ingested carbamates in our study varied in chemistry, we surprisingly did not find any of the marked variation in pattern of clinical presentation we have noted with OP insecticides of similarly varied chemistry.[16] These carbamates appear representative of the carbamate class as a whole, having oral rat LD_50_s and log *P*s across the class range (Figure 6). The differences in proportion of patients dying and requiring ventilation is likely explained in part by their varying formulation which affected the dose ingested and median plasma concentration. Patients appeared to find it more difficult to ingest a large dose of the 3% wettable powder formulation of carbofuran compared with the liquid formulations of the other two carbamates. The high case fatality caused by carbosulfan was likely due to the production of carbofuran after ingestion. The rat LD_50_ did not accord with human toxicity.

Few patients became sick after 12 h, suggesting that the intermediate syndrome is uncommon after carbamate poisoning. This conclusion differs from an Indian study of 66 patients with isolated carbosulfan poisoning in which nine (13.6%) died and 12 (18.2%) had respiratory failure after 12 h.[25] Persistent respiratory failure occurred in four carbofuran-poisoned patients as they regained consciousness with resolution of the acute cholinergic crisis,[25] suggesting NMJ failure that developed during the cholinergic crisis (as has been reported with OP poisoned patients [17]). The different, higher-toxicity, liquid EC formulation of carbofuran in India might be responsible for this difference.

The median duration of intubation for survivors was 67.8 h. This contrasts with the median duration in a case series of OP-poisoned patients intubated more than 24 h post-ingestion (219 h) but is more similar to the median 33 h intubation noted for OP-poisoned patients intubated within 24 h.[17] It is unclear whether treatment with oximes would have reduced the duration of intubation but pralidoxime did not do so in a clinical trial of OP-poisoned patients we performed at the same time.[26] The long duration intubations were required to treat sepsis and pneumonia in patients who had often aspirated.

Seizures were uncommon in this case series; they were, however, most common after poisoning with the most fat-soluble carbamate (carbosulfan), similar to the situation with OP insecticides.[15,16]

Lower GCS on admission was associated with worse outcome; of note, 30% of deaths after carbosulfan poisoning occurred in patients who presented with normal consciousness. This was uncommon with the other carbamates, consistent perhaps with the time taken to convert carbosulfan to the more toxic carbofuran after ingestion. Out-of-hospital deaths were not recorded in this study and it is plausible that this delay also meant these were less likely with carbosulfan. A low GCS (≤13/15) was previously found to be an independent predictor of complications in a small retrospective case series of 52 patients poisoned by WHO Toxicity Class Ib and II carbamates.[27] These same authors found that deaths after carbamate poisoning occurred in patients presenting to hospital in cardiorespiratory arrest, a situation that was uncommon for the OP insecticides.[23] This early presentation of severe carbamate poisoning may be due to these insecticides not requiring activation after absorption.[11,12]

Current treatment for carbamate-poisoned patients, as for OP insecticide-poisoned patients, is only partially effective, with case fatality for carbosulfan-poisoned patients greater than 10%. The lack of variability in clinical syndrome suggests that there is no need to develop management approaches specific to particular carbamate compounds. However, the poor outcome indicates that better therapies are required or that carbamates should not be used in small-scale agriculture in rural communities where safe use due to limited agricultural and economic resources is not possible.

## Limitations

Limitations of this study include the lack of a blood sample from all patients to identify the carbamate ingested and the lack of facilities for routine measurement of acetylcholinesterase activity. Plasma samples were available for 198 patients and a carbamate could be detected in 83.8% of them, suggesting that the history effectively identified the ingested carbamate. We did not exclude patients without carbamate in the blood from the analysis since we did not have blood samples for all patients and this would have introduced bias.

Post-mortem data were not available for confirmation of the cause of death. Cause of death was decided on clinical grounds.

All patients were seen and treated in a resource-limited hospital. Intensive care beds were limited with no specialist intensivists and the use of only dopamine/dobutamine for cardiovascular support. It is likely that some of these patients would have survived if treated in a world-class intensive care unit. There has also been a long lag time between data collection and publication; it is possible that improvements in intensive care provision in these hospitals might have improved outcome. However, the majority of patients with carbamate self-poisoning present to such busy resource-limited district hospitals and so the data reported here represent well the global situation.

Dimethoate poisoning has been shown to be influenced by ethanol co-ingestion/intoxication and by blood ethanol concentration [28] and also by the solvent in a large animal model.[29] We noted no evidence of an effect of ethanol or solvent in this study but unfortunately were not able to look for variation in, and concentration of, different solvents and ethanol. Such studies are required to better understand all forms of pesticide poisoning.

## Conclusion

We found that carbamate self-poisoning showed surprisingly little variation in the clinical pattern of poisoning and, for liquid emulsion concentrates of WHO Class II compounds, case fatalities as high as, and often higher than, OP insecticides of similar toxicity. Carbamates do not appear to be less toxic than OP insecticides in human poisoning. Regulation of agricultural pesticides that encourages farmers to switch from OP to carbamate insecticides will likely not result in major global reduction in suicide. However, reformulation of hazardous pesticides from high-concentration liquid formulations to low-concentration solid preparations should reduce deaths.
